# Possible Earlier Diagnosis of Ulcerative Colitis-Associated Neoplasia: A Retrospective Analysis of Interval Cases during Surveillance

**DOI:** 10.3390/jcm10091927

**Published:** 2021-04-29

**Authors:** Takashi Hisabe, Toshiyuki Matsui, Kazutomo Yamasaki, Tsuyoshi Morokuma, Kenmei Aomi, Naoyuki Yoshizawa, Noritaka Takatsu, Kenshi Yao, Toshiharu Ueki, Kitaro Futami, Hiroshi Tanabe, Akinori Iwashita

**Affiliations:** 1 Department of Gastroenterology, Fukuoka University Chikushi Hospital, Chikushino, Fukuoka 818-8502, Japan; matsui@fukuoka-u.ac.jp (T.M.); ky875416@yahoo.co.jp (K.Y.); morokuma1972@yahoo.co.jp (T.M.); yoshiaki1979@aom.jp.net (K.A.); nanataku4711@yahoo.co.jp (N.Y.); kouta-yu@cap.bbiq.jp (N.T.); tosiueki@fukuoka-u.ac.jp (T.U.); 2 Department of Endoscopy, Fukuoka University Chikushi Hospital, Chikushino, Fukuoka 818-8502, Japan; yao@fukuoka-u.ac.jp; 3 Department of Surgery, Fukuoka University Chikushi Hospital, Chikushino, Fukuoka 818-8502, Japan; kitaro@fukuoka-u.ac.jp; 4 Department of Pathology, Fukuoka University Chikushi Hospital, Chikushino, Fukuoka 818-8502, Japan; h.tanabe197265@gmail.com (H.T.); iwa-aki@fukuoka-u.ac.jp (A.I.)

**Keywords:** ulcerative colitis, colorectal cancer, ulcerative colitis-associated neoplasia, surveillance, interval cancer

## Abstract

Background: Early detection of ulcerative colitis-associated neoplasia (UCAN) is often difficult. The aim of this study was to clarify the morphology of initial UCAN. Methods: White-light colonoscopy images obtained within the 2 years before UCAN diagnosis were retrospectively reviewed. The primary endpoint was the frequency of visible or invisible neoplasia on the endoscopic images before UCAN diagnosis. The secondary endpoints were comparisons of (1) visible or invisible neoplasia on initial endoscopic images of early-stage and advanced cancers, (2) the clinical backgrounds of patients in whom neoplasia was visible or invisible on initial endoscopic images, and (3) the clinical backgrounds of patients with distinct and indistinct UCAN borders. Results: Of the 27 UCAN lesions (11 early-stage; 16 advanced-stage), 25.9% (*n* = 7) were initially visible and 74.1% (*n* = 20) were invisible. The mean interval between the last surveillance colonoscopy and UCAN diagnosis was 14.5 ± 6.7 months. Of early-stage cancers, 18.2% (*n* = 2) were visible and 81.8% (*n* = 9) were invisible. Of advanced-stage cancers, 31.3% (*n* = 5) were visible and 68.8% (*n* = 11) were invisible. Invisible lesions were significantly more common in the rectum (*p* = 0.011) and tended to be more common in patients with inflammation and left-sided colitis (*p* = 0.084, *p* = 0.068, respectively). Patients with indistinct UCAN borders were significantly more likely to present with inflammation than those with distinct UCAN borders (*p* = 0.021). Conclusion: More careful surveillance is needed because rectum lesions and inflammation are difficult to identify as neoplasia even within the 2 years before a UCAN diagnosis.

## 1. Introduction

The frequency of colorectal cancer is significantly higher in patients with ulcerative colitis (UC) than in the general population [1] because of chronic inflammation of the intestinal tract in the former group. In recent years, the increased number of patients with UC and the prolongation of disease durations have emphasized the need for surveillance of UC-associated neoplasia (UCAN). Researchers have proposed the dysplasia–carcinoma sequence theory as the mechanism underlying the development of inflammatory bowel disease (IBD)-related cancers and have reported the frequent involvement of *TP53* mutations [2,3]. In contrast, these cancers less frequently involve *APC* and *KRAS* mutations [4,5]. Abnormalities in mismatch repair genes and CpG island methylation [6,7,8] are observed in early-stage disease and are considered to promote progression from a low- to high-grade dysplasia and subsequent carcinogenesis. In summary, research has gradually revealed the pathology of UCAN, including the potential risk factors.

Surveillance colonoscopy is recommended for the early detection of IBD-related cancers [9,10,11], and reports suggest that appropriate surveillance measures contribute to reductions in the incidence of colorectal cancer and the associated mortality [12]. However, early detection is difficult in patients with IBD, even if surveillance colonoscopy is performed periodically. In a previous report, 5% of patients who underwent colonoscopy every 1–2 years developed colorectal cancer, and half of these patients had interval cancer [13]. Therefore, the characteristic endoscopic findings of early-stage lesions must be clarified to avoid further missed lesions. A detailed analysis of the endoscopic findings during surveillance should reveal noteworthy findings. In this study, therefore, we retrospectively reviewed the white-light colonoscopy images obtained from patients before a diagnosis of UCAN and examined the initial UCAN lesions to clarify the clinicopathological features.

## 2. Materials and Methods

### 2.1. Subjects

This single-center retrospective observational study was approved by the ethics committee of Fukuoka University. The study was performed in accordance with the ethical principles outlined in the Declaration of Helsinki.

The following inclusion criteria were applied during the patient selection process: (1) a neoplastic lesion identified by colonoscopy, subsequent surgical resection, and histopathological diagnosis of UCAN (early-stage or advanced-stage colorectal cancer) at Fukuoka University Chikushi Hospital between January 2000 and December 2019 and (2) the identification of lesion sites by reviewing the last surveillance white-light colonoscopy images obtained within the 2 years before the UCAN diagnosis. The following exclusion criteria were also applied: (1) a diagnosis of sporadic cancer or suspected sporadic cancer; (2) a lack of evaluable endoscopic images because of poor observation conditions and inadequate bowel preparations; and (3) a lack of confirmation of the clinical backgrounds of a patient.

### 2.2. Study Procedures and Endpoints

All white-light colonoscopy static images, medical records, and pathology records were analyzed retrospectively. The concordance of the location of UCAN was evaluated by identifying each specific index (e.g., folds, polyps, and strictures). A visible neoplasia was defined as a neoplasia that could be visualized at white-light colonoscopy and an invisible one was defined as a neoplasia that could not be visualized at white-light colonoscopy. A distinct UCAN border was defined as a lesion’s border that was discrete and could be distinguished from the surrounding mucosa. An indistinct UCAN border was defined as a lesion’s border that was not discrete and could not be distinguished from the surrounding mucosa. The final evaluation of visible or invisible as neoplasia and distinct or indistinct UCAN borders were decided by the consensus agreement of at least two reviewers who had each performed more than 3000 colonoscopies (T.H., T.M., K.Y., T.M., K.A., or N.Y.). In our facility, all the colonoscopy images are saved as a serial picture so that we can see the part of the colon where the image was taken.

All participants had a confirmed UC diagnosis, which was based on clinical, endoscopic, radiological, and histological criteria. The macroscopic type of each lesion was described according to the Endoscopic and Chromoendoscopic Atlas [14]. The Mayo scores for endoscopic mucosal findings (i.e., Mayo endoscopic subscore) [15], in which 0 was defined as remission and >1 as active, were used to determine the index of endoscopic activity. All resected specimens were fixed in a 20% buffered formalin solution. All lesions were embedded on paraffin and cut into 2 to 3 mm slices. Each section was subsequently stained for hematoxylin and eosin, p53, and Ki67. Histopathological diagnosis was performed by an experienced board-certified pathologist (A.I.). pTis and pT1 lesions were defined as early-stage cancers, and pT2, pT3, and pT4 lesions were defined as advanced-stage cancers. The histopathological diagnosis was used as the gold standard.

The primary endpoint was the frequency of visible or invisible neoplasia on the endoscopic images before UCAN diagnosis. The secondary endpoints were (1) a comparison of visible or invisible neoplasia on the initial endoscopic images of early-stage and advanced cancers, (2) a comparison of the clinical backgrounds of patients whose initial endoscopic images contained visible or invisible neoplasia, and (3) a comparison of the clinical backgrounds of patients with distinct and indistinct UCAN borders.

### 2.3. Statistical Analysis

Categorical variables were compared using Fisher’s exact test. *p* values < 0.05 were considered statistically significant. SPSS version 21J (SPSS, Chicago, IL, USA) was used for all statistical analyses.

## 3. Results

A total of 53 UCAN patients were identified between January 2000 and December 2019 for inclusion in the study. A total of 34 UCAN patients had a colonoscopy performed within the 2 years before the UCAN diagnosis. Of these, 7 UCAN patients where we could not detect the lesion sites by reviewing the last surveillance colonoscopy images and 1 with a lack of the clinical backgrounds were excluded. A total of 27 lesions in 26 UCAN patients that met the criteria were analyzed. The male:female ratio of the patients was 18:8, and the mean age at UCAN diagnosis was 49.3 ± 13.3 years. The mean disease duration was 15.5 ± 6.2 years. Regarding the clinical courses, 10 patients had the relapse remitting type, whereas 16 had the chronic continuous type. Eighteen and eight patients had extensive colitis-type and left-sided colitis-type, respectively. The purpose of colonoscopy was surveillance in more than 80% of cases. Even in non-surveillance colonoscopy cases, a detailed colonoscopy was performed, including detection of UCAN. Eight UCAN lesions were located in the colon and 19 were located in the rectum. Eleven early-stage cancer lesions and 16 advanced-stage cancer lesions were identified (Table 1).

**Figure 1:**
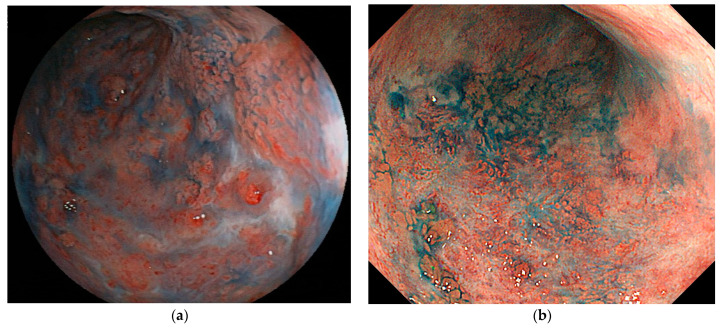
A visible lesion that progressed to early-stage cancer. (**a**) An image obtained 12 months before cancer diagnosis reveals background mucosa in the active phase and slightly elevated lesions with indistinct border in the sigmoid colon. Although the tumor was recognized, the biopsy revealed no neoplastic changes and colonoscopy was performed every few months. (**b**) At the time of the cancer diagnosis, the background mucosa was in the remission phase and slightly elevated lesions with indistinct border was observed. Histopathological findings indicated a well-differentiated adenocarcinoma, pTis.

**Figure 2:**
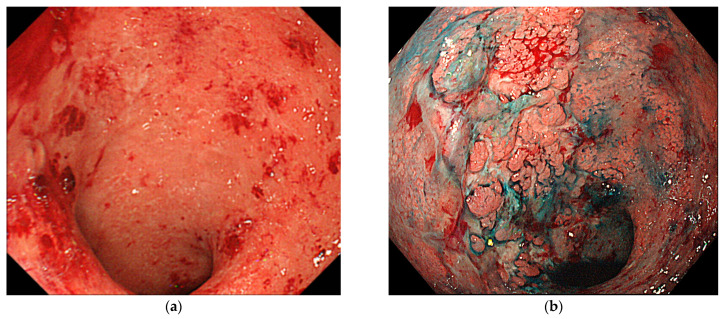
An invisible lesion that progressed to early-stage cancer. (**a**) At 14 months before the cancer diagnosis, the background mucosa was in the active phase and finely granular mucosa was observed in the Ra. A biopsy was not performed. (**b**) At the time of cancer diagnosis, the background mucosa was in the active phase, and a villous and granular elevated lesion and an ulcer were visible. The histopathological findings indicated a well-differentiated adenocarcinoma, pTis.

**Figure 3:**
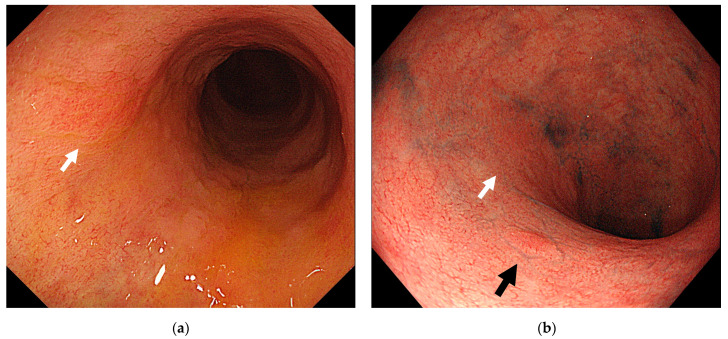
An invisible lesion that progressed to early-stage cancer. (**a**) At 14 months before the cancer diagnosis, the background mucosa was in the remission phase, and a red, flat lesion (white arrow) was observed in the Ra. A biopsy of the same site showed low grade dysplasia. (**b**) At the time of cancer diagnosis, superficial elevated lesion with distinct border (black arrow) was observed. Histopathological findings indicated a well-differentiated adenocarcinoma, stage pTis.

**Figure 4:**
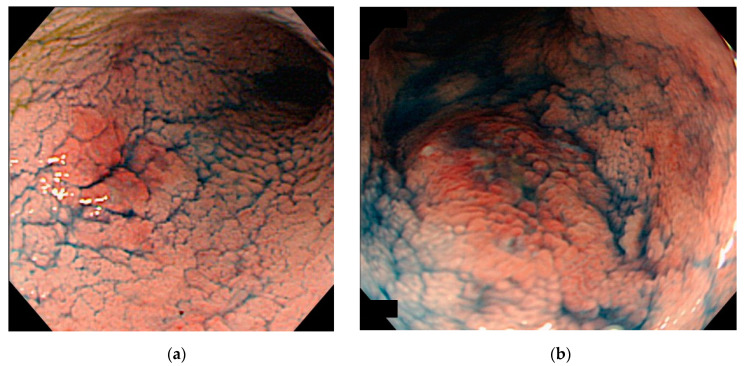
A visible lesion that progressed to advanced-stage cancer. (**a**) At 17 months before the cancer diagnosis, the background mucosa was mildly active, and slightly elevated lesion was observed in the sigmoid colon. Although the biopsy revealed tubular adenoma, medical treatment was intensified because of the exacerbation of UC. (**b**) At the time of cancer diagnosis, the background mucosa was in the mildly active phase, and elevated lesion with indistinct border was observed. The histopathological findings indicated a well to moderately differentiated adenocarcinoma, pT4.

**Figure 5:**
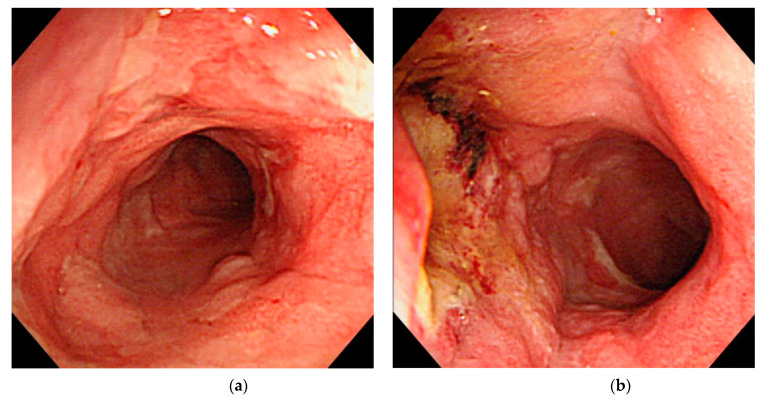
An invisible lesion that progressed to advanced-stage cancer. (**a**) At 15 months before the cancer diagnosis, the background mucosa was in remission. (**b**) At the time of cancer diagnosis, the background mucosa was in the remission, and an ulcer in the Rb and irregular elevated lesion with indistinct border in the Rb were observed. The histopathological findings indicated an endocrine cell carcinoma, pT4.

The mean interval between the last surveillance colonoscopy and the UCAN diagnosis was 14.5 ± 6.7 months. Of the 27 UCAN lesions, 25.9% (*n* = 7) were visible as neoplasia and 74.1% (*n* = 20) were invisible as neoplasia on the initial endoscopic images obtained before the diagnosis of UCAN. Of the 11 early-stage cancer lesions, 18.2% (*n* = 2) were visible as localized lesions (Figure 1) and 81.8% (*n* = 9) were invisible as neoplasia on initial images obtained during a mean interval of 12.9 ± 7.4 months before a UCAN diagnosis (Figure 2 and Figure 3). Of the 16 advanced-stage cancer lesions, 31.3% (*n* = 5) were visible as neoplasia (Figure 4) and 68.8% (*n* = 11) were invisible as neoplasia (Figure 5) on initial images obtained during a mean interval of 15.4 ± 6.2 months prior to UCAN diagnosis (Table 2). Of the seven cases determined to be visible on the review of the endoscopic images, five cases were recognized as tumors at the previous colonoscopy. Three of these cases were not diagnosed as neoplasia by biopsy and two of these were diagnosed as adenomas, but medical treatment of UC was the priority.

A comparison of the clinical backgrounds of patients whose initial endoscopic images were visible or invisible as neoplasia revealed that a significantly greater number of lesions in the rectum were invisible, compared to those in the colon (*p* = 0.011). Furthermore, patients with active endoscopic inflammation and left-sided colitis were more likely to have lesions that were invisible as neoplasia (*p* = 0.084 and *p* = 0.068, respectively; Table 3). In addition, 50% (4/8) of the colon and 63.2% (12/19) of the rectum were in the active stage (*p* = 0.675). Finally, patients with indistinct UCAN borders were significantly more likely to present with active endoscopic inflammation within the 2 years before the diagnosis of UCAN, compared to those with distinct UCAN borders (*p* = 0.021; Table 4).

## 4. Discussion

In this retrospective review of white-light colonoscopy images obtained within the 2 years before a diagnosis of UCAN, we determined that up to 74.1% of lesions were not identified as neoplasia. In addition, 81.8% of patients with early-stage cancers were not identified during a mean interval of 12.9 months, whereas 68.8% of patients with advanced-stage cancer were not identified during a mean interval of 15.4 months. In our previous multicenter questionnaire survey [16] on the endoscopic findings obtained within the 3 years before a diagnosis of UCAN, we inquired about the endoscopic findings from 54 UCAN lesions. In that survey, 60.0% of early stage cancers were not identified as neoplasia after a mean follow-up period of 38.6 months until the diagnosis of UCAN, whereas 57.9% of advanced-stage cancers were not identified after a mean follow-up period of 32.8 months until the diagnosis of UCAN. Although we set a relatively shorter observation period in this study, many cases still remained unidentified as neoplasia.

The difficulty in detecting UCAN at an early stage and the diversity of the endoscopic appearances of these lesions are attributed to differences in the histological features relative to those of sporadic tumors. UCANs are often poorly circumscribed relative to the surrounding mucosa because of modifications caused by active or chronic background inflammation and a lack of change in the surface layer caused by bottom-up tumor formation (from a deep to middle glandular layer). In this study, significantly more patients with indistinct UCAN borders exhibited active endoscopic inflammation within the 2 years before the diagnosis of UCAN. Our observation further supports an indistinct UCAN border as a factor that increases the difficulty of early cancer detection. We therefore believe that the early detection of cancers would be enhanced by more effective control of disease activity.

Several reports have described lesion miss rates during colonoscopy. In a surveillance study of elderly patients with IBD, Wang et al. [17] estimated that 15.8% of early-stage cancers in patients with UC were missed by colonoscopies performed within the 36 months before lesion detection. Rutter et al. [13] reported that 30 of 600 (5%) patients with UC who underwent surveillance colonoscopy every 1–2 years developed colorectal cancer. Sixteen of those 30 patients had interval cancers, among whom 13 were found to have advanced cancers. Interval cancers may account for approximately 50% of the cancers identified during IBD surveillance. It is highly likely that missed lesions represent a major contribution to the development of interval cancers [18].

As mentioned previously, IBD-related cancers are often detected at an advanced stage, as early detection is difficult. However, a subsequent study by Choi et al. [19] indicated that the incidence rate of interval cancer per decade had decreased significantly over the past four decades, from 2.5 per 1000 patient years in the first decade to 0.4 per 1000 patient years in the fourth decade. In contrast, the authors found that the incidence rate of UC-associated dysplasia was significantly increased in the fourth decade relative to the third decade (11.3 vs. 6.3 per 1000 patient years). These results suggest that recent improvements in colonoscopy image quality, chromoendoscopy, and image-enhanced endoscopy as well as the quality of colonoscopy examinations have enabled the earlier detection of dysplasia and early-stage cancers, consistent with the findings of other reports [20,21]. Despite these advances, a questionnaire survey of 541 physicians who attended IBD meetings in Japan [22] revealed that only 49% of the participants used chromoendoscopy, 24% used a magnifying endoscope, and 8% used narrow-band imaging observations in addition to white-light observations. Basically, clinical surveillance remains dependent on white-light colonoscopy. Therefore, a review of the white-light colonoscopy images and retrospective analysis of morphological changes are very important means of clarifying initial images of UCAN and identifying findings that would facilitate early detection.

In this study, lesions that were not identified by colonoscopy within the 2 years before a diagnosis of UCAN were significantly more commonly detected in the rectum than in the colon. Eluri et al. [23] investigated the prevalence of high-grade dysplasias or cancers that were found during colectomies but had remained undetected during previous colonoscopies; in that study, 84% of the lesions were observed in the rectum, consistent with our data. Structurally, the rectum, particularly the lower rectum, often represents a dead angle with respect to endoscopic observation. Therefore, a retroflexion technique may be important for observations near the dentate line. Additionally, of the 17 rectal lesions in our study that were invisible as neoplasia on initial images, 12 were associated with active endoscopic inflammation. There was no statically significant difference, but there was more active inflammation in the rectum than in colon, which would likely make it difficult to identify neoplasia. The rectum should be observed in particular with other methods such as chromoendoscopy and image-enhanced endoscopy (narrow-band imaging, blue laser imaging) in addition to the conventional observation.

According to a previous report, UCAN was detected significantly earlier and the overall survival rate was better in patients whose lesions were detected by surveillance than in those whose lesions were not detected by surveillance [24]. Various countries have proposed surveillance guidelines and most have been based on random biopsy. However, this method yields a low lesion detection rate and is not efficient in terms of the testing time and medical costs [25]. Consequently, targeted biopsy via chromoendoscopy has entered the mainstream in recent years. A Japanese randomized controlled trial [26] that compared random and targeted biopsies found no significant differences in the number of dysplasias detected per colonoscopy.

Additionally, surveillance colonoscopy should be performed when the disease is in the remission phase to avoid a potential misdiagnosis between inflammatory changes and dysplasia [27,28]. Although patients with active endoscopic inflammation face a high risk of UCAN [29], our data included many lesions that could not be identified as neoplasia in the active phase. This observation emphasizes that many lesions at the site of inflammation cannot be identified as neoplasia and thus both a random biopsy and detailed observation should be considered in such cases. In recent years, each surveillance guideline has set the interval according to the risk of UCAN occurrence. Currently, this interval is set to every 3 years for patients at an intermediate risk and every 5 years for those at a low risk. However, in our study, several advanced cancers were missed even when patients were screened at intervals of 2 years. Four of six lesions that were invisible as neoplasia were identified at an advanced stage, despite the maintenance of remission. A longer interval could decrease the effectiveness of surveillance and may result in an increased incidence of interval cancers. Therefore, we believe it necessary to investigate further whether the surveillance interval could be extended according to the risk of UCAN.

This study had several limitations. First, this was a retrospective study of a limited number of patients at a single institution, and the study period spanned approximately 20 years. This likely led to time-related differences in the patients’ clinical backgrounds, and several experienced endoscopists performed surveillance colonoscopies. Nonetheless, a review of the accumulated images of individual patients and comparison of morphological changes over time would be needed to clarify the natural history of these tumors. Moreover, the quality of colonoscopy image has improved. However, similar results obtained in cases using recent high-definition colonoscopes and the visible rate did not differ between the first and second half of the study period. The image quality may not be the only factor that is making it difficult to detect UCANs. Second, this study included a larger number of rectal lesions than colon lesions. In Japan, 51% of UCANs arise in the rectum and this imbalance contributes to the status of the rectum as the most commonly affected site [24]. Compared to the rectum, it is often difficult to identify the same site in the colon during an image review. Therefore, we cannot rule out bias in the case selection process.

## 5. Conclusions

Our results suggested that more careful surveillance, including random biopsies and chromoendoscopy/image-enhanced endoscopy, is needed at sties of active inflammation in the rectum.

## Figures and Tables

**Table 1:** Clinicopathologic characteristics of the patients.

Number of patients	26
Number of lesions	27
Gender (male/female)	18/8
Age (mean ± SD)	49.3 ± 13.3 years
Duration of disease (mean ± SD)	15.5 ± 6.2 years
Clinical course	
Relapse remitting type	10 (38.5%)
Chronic continuous type	16 (61.5%)
Type of disease	
Left sided colitis	8 (30.8%)
Extensive colitis	18 (69.2%)
Reason for colonoscopy	
At the time of UCAN diagnosis	
Surveillance	21 (80.8%)
Abdominal pain	4 (15.4%)
Bloody stool	1 (3.8%)
Before UCAN diagnosis	
Surveillance	23 (88.5%)
Abdominal pain	1 (3.8%)
Bloody stool	2 (7.7%)
Lesion location	
Ascending colon	1 (3.7%)
Transverse colon	0 (0%)
Descending colon	2 (7.4%)
Sigmoid colon	5 (18.5%)
Rectum	19 (70.4%)
Macroscopic type	
Protruted	12 (44.4%)
Slightly elevated	9 (33.3%)
Flat	1 (3.7%)
Depressed	2 (7.4%)
Mixed	3 (11.1%)
Histological findings	
pTis, pT1	11 (40.7%)
pT2, pT3	16 (59.3%)
Treatment	
At the time of UCAN diagnosis	
5-aminosalicyclic acid	23/26 (88.5%)
Immunomodulator	8/26 (30.8%)
Corticosteroid	5/26 (19.2%)
Biologics	3/26 (11.5%)
Before UCAN diagnosis	
5-aminosalicyclic acid	23/26 (88.5%)
Immunomodulator	8/26 (30.8%)
Corticosteroid	6/26 (23.1%)
Biologics	3/26 (11.5%)
Primary sclerosing cholangitis	0
Family history colorectal cancer in first degree relative	0

SD, standard deviation; UCAN, ulcerative colitis-associated neoplasia.

**Table 2:** The frequency of visible or invisible as neoplasia before UCAN diagnosis.

	Surveillance Interval (Mean ± SD)	Visible Lesions	Invisible Lesions
Total (*n* = 27)	14.5 ± 6.7 months	25.7% (7/27)	74.1% (20/27)
pTis, pT1 (*n* = 11)	12.9 ± 7.4 months	18.2% (2/11)	81.8% (9/11)
pT2, pT3 (*n* = 16)	15.4 ± 6.2 months	31.3% (5/16)	68.8% (11/16)

SD, standard deviation.

**Table 3:** Comparison of the clinical backgrounds of patients whose initial endoscopic images were visible or invisible.

	Visible Lesions (*n* = 7)	Invisible Lesions (*n* = 20)	*p*-Value
Clinical course			
Relapse remitting type	2	8	0.678
Chronic continuous type	5	12	
Endoscopic disease activity			
Active	2	14	0.084
Remission	5	6	
Type of disease			
Extensive colitis	7	12	0.068
Left sided colitis	0	8	
Macroscopic type			
Protruted, Mixed	5	10	0.408
Slightly elevated, Flat, Depressed	2	10	
Lesion location			
Colon	5	3	0.011
Rectum	2	17	
Histological findings			
Tis, T1	2	9	0.662
T2, T3	5	11	
Colonoscopy period			
First half (2000–2009)	3	8	1.000
Second half (2010–2019)	4	12	

**Table 4:** Comparison of the clinical backgrounds of patients with distinct and indistinct UCAN borders.

	Distinct Border (*n* = 14)	Indistinct Border (*n* = 13)	*p*-Value
Clinical course			
Relapse remitting type	6	4	0.695
Chronic continuous type	8	9	
Endoscopic disease activity			
Active	4	11	0.021
Remission	10	2	

## Data Availability

The datasets used and analyzed during the current study are available from the corresponding author on reasonable request.
